# Use of a hydrophilic coating wire reduces significantly the rate of central vein punctures and the incidence of pneumothorax in totally implantable access port (TIAP) surgery

**DOI:** 10.1186/s12893-017-0329-4

**Published:** 2017-12-07

**Authors:** Georgios Polychronidis, Roland Hennes, Cosima Engerer, Phillip Knebel, Daniel Schultze, Thomas Bruckner, Beat P. Müller-Stich, Lars Fischer

**Affiliations:** 1 Department of Surgery, University of Heidelberg, INF 110, 69120 Heidelberg, Germany; 2 Department of Medical Biometry, Institute of Medical Biometry and Informatics (IMBI), Im Neuenheimer Feld 130.3, 69120 Heidelberg, Germany; 3 Department of Surgery, Klinikum Mittelbaden Baden-Baden Bühl, Balger Str. 50, 76532 Baden-Baden, Germany

**Keywords:** Totally implantable access port, Pneumothorax, Guide wire, Venous cut-down

## Abstract

**Background:**

Insertion of a Totally Implantable Access Port (TIAP) can be performed either via Central Vein Puncture (CVP) or Brachiocephalic Vein Cut-down (venous section-VS). The primary success rate of TIAP implantation using VS rarely ever achieves 100%. The objective of this study was to describe a modified VS approach using a hydrophilic coated wire (TVS).

**Methods:**

From 01.01.2015 to 31.12.2015, all patients receiving TIAP implantations were screened. During this time, all patients in whom the primary VS procedure was found to be unsuccessful were analysed.

**Results:**

In 2015, 1152 patients had TIAP implantations performed by 24 different surgeons. Of these, 277 patients needed a second line rescue strategy either by CVP (*n* = 69) or TVS (*n* = 208). There were no statistically significant differences regarding demographics or indication for TIAP implantation between CVP and TVS. The operation time and the qualification of the operating surgeon between CVP and TVS did not differ significantly. After the introduction of the guidewire with a hydrophilic coated wire, the need for CVP decreased significantly from 12.7% to 8.8% (*p* < 0.0001). In patients receiving CVP as a second line rescue strategy, the incidence of pneumothorax (*n* = 3 patients (4.3%)) was significantly higher compared to patients with TVS as a second line rescue strategy (*n* = 1 patient (0.48%), *p* = 0.02).

**Conclusion:**

The use of a hydrophilic coated wire significantly decreased the number of CVP and the incidence of pneumothorax. TVS is a safe and successful second-line rescue strategy.

## Background

Since the first insertion of a Totally Implantable Access Port (TIAP) in 1982, the use of this central venous access system has increased dramatically over the last years [1]. As an example, the total amount of implanted TIAPs in Germany was over 125,000 in 2014 [2, 3]. Nowadays, TIAPs are not only used for chemotherapy in cases of malignant diseases, but also for blood and blood products transfusion, as well as, for the administration of parenteral nutrition [1–7].

**Table 1:** Baseline Characteristics of Analysed Patients

	All Patients n (%)	Central Vein Puncture as a second line rescue strategy n (%)	Use of guidewire with a hydrophilic coating as a second line rescue strategy n (%)
n	277	69	208
Mean Age	58.7 years (SD ±15.1)	58.6 years (SD ±16.4)	58.7 years (SD ±15.1)
Female Patients	149 (53.7%)	38 (55.1%)	111 (53.4%)
Indication for TIAP Implantation			
Gastrointestinal Malignancy	84 (30.3%)	19 (27.5%)	65 (31.2%)
Gynecological Malignancy	74 (26.7%)	20 (29.0%)	54 (26.0%)
Hematological Malignancy	59 (21.3%)	15 (21.7%)	44 (21.2%)
Other Malignancies/Reasons	60 (21.7%)	15 (21.7%)	45 (21.6%)
Patients with first TIAP implantation	245 (88.4%)	58 (84.1%)	187 (89.9%)
TIAP implantation on the patients’ left side	220 (79.4%)	58 (84.1%)	163 (78.4%)
Central Vein Puncture	100 (36.1%)	69 (100%)	31 (14.9%)

Abbreviations: *SD* = standard deviation, *TIAP* = Totally Implantable Access Port

**Table 2:** Intra- and postoperative data of Totally Implantable Access Port (TIAP) implantation using either Central Vein Puncture (CVP) or modified vein cut-down using hydrophilic coated guide-wire (TVS) as a second line rescue strategy

	All Patients n (%)	Central Vein Puncture as a second line rescue strategy n (%)	Use of guidewire with a hydrophilic coating as a second line strategy n (%)	p*
n	277	69	208	
Mean operation time (SD)	37.7 min (± 20.9 min)	35.6 min (± 16.8 min)	38.4 min (± 22.2 min)	0.347
Operation performed by Resident alone (n)	76 (27.5%)	16 (23.2%)	60 (28.8%)	0.351
Operation performed by Attending alone (n)	171 (61.7%)	45 (65.2%)	126 (60.6%)	0.568
Operation performed by Resident and Attending	30 (10.8%)	8 (11.6%)	22 (10.6%)	0.814
Early Morbidity				
Intraoperative bleeding	3 (1%)	0 (0%)	3 (1%)	0.316
Pneumothorax	4 (1.4%)	3 (4.3%)	1 (0.5%)	0.020
Need for thorax drainage	1 (0.4%)	1 (1.4%)	0 (0%)	0.082
Late Morbidity				
Thrombosis	2 (0.7%)	0 (0%)	2 (0.9%)	0.414
TIAP Infection	17 (6.1%)	3 (1.4%)	14 (6.7%)	0.475
Sepsis due to TIAP Infection	14 (5%)	2 (2.9%)	12 (5.7%)	0.346
Non-Function due to dislocation	8 (2.9%)	2 (2.9%)	6 (2.9%)	0.995
Pinch-off syndrome	1 (0.4%)	0 (0%)	1 (0.5%)	0.564
Postoperative hematoma	2 (0.7%)	0 (0%)	2 (0.9%)	0.414
Removal of TIAP	26 (9.4%)	6 (8.7%)	20 (9.6%)	0.803

Abbreviations: SD = standard deviation, TIAP = Totally Implantable Access Port, CVP = Central Vein Puncture, TVS = vein cut down using a guidewire with a hydrophilic coating; Terumo® Venae Sectio

*p value determined according to the Kruskal Wallis Test for continuous variables and Chi-squared Test for dichotomous variables

There are two main approaches used to implant a TIAP. Firstly, via the direct puncture of a central vein (CVP) and secondly, through a classical cut-down of the brachiocephalic vein (CVS). In recent years, the CVS was adapted by using a guide-wire in a modified Seldinger technique (MVS) [8, 9]. Both main approaches, i.e. the CVP and CVS or the MVS, can be performed with low morbidity [10, 11]. However, the risk of pleural injury with consecutive development of a pneumothorax will always exist when CVP is utilised [7, 12]. On the other hand, the success rate of TIAP implantation via CVS/MVS will never reach 100% [10, 13–17], since there will be patients in whom CVS/MVS cannot be performed because of the absence of a brachiocephalic vein or the presence of one that is inadequate sized. Another reason for an unsuccessful TIAP implantation by CVS/MVS is a steep junction of the brachiocephalic vein into the central venous system) [18–21].

Although the use of a guide-wire as a means of dealing with a situation in which the catheter cannot be inserted into the cephalic vein is a basic part of the MVS [22], there have not been any specifications regarding the kind of guide-wire to be used. However, clinical data derived mostly from interventional radiologist series suggest that there are guide-wires available that are either particularly suited for small vessels or able to pass through steep junctions of blood vessels [23]; the widely used Terumo® Radifocus Guidewire M Standard (Terumo® wire) type is one of these [24]. The Terumo® wire is a guide-wire with a hydrophilic polymer coating, thus allowing for low friction manoeuvrability. The aim of this study was to examine the impact of this guide-wire (TVS) as a second line rescue strategy in patients with primary TIAP implantations.

## Methods

This retrospective study was approved by the local ethics committee of the University of Heidelberg (S-584/2016). The study was conducted at the outpatient clinic of the General Surgery Department, University of Heidelberg. Funding was not provided.

### Participants

**Fig. 1:**
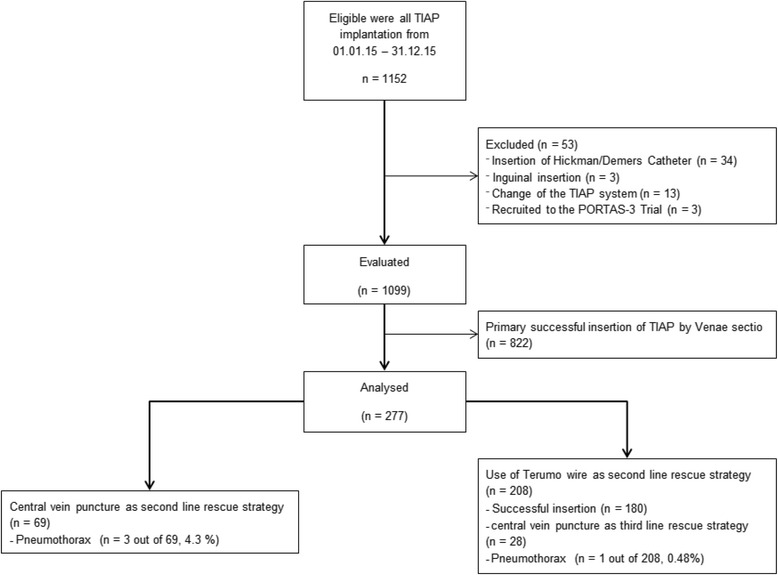
Flowchart of this retrospective study

The criterion for this study was to focus on all elective TIAP insertions for either benign or malignant diseases that were performed under local anaesthesia throughout 2015. A Flowchart of this retrospective study is shown in Fig. 1. Based on previously published data [19] a follow-up period of 6 months was established as sufficient in order to detect possible complications after TIAP implantation. Complications during the first 30 days after TIAP insertion were classified as early, while complications presenting after that time were classified as late complications.

### Interventions

The principal techniques for CVP, CVS, and MVS, respectively, have been described in detail in previous publications [22, 25]. The choice of the implantation side was predominantly left to the patients’ discretion. In general, all patients were positioned on the operating table in a five-degree, reverse Trendelenburg’s position. The neck, chest, and shoulders of the patients were prepared and draped in a sterile manner. Antibiotic prophylaxis was given in cases where patients were at risk for endocarditis (in accordance with the local standards) or only if a TIAP was to be used for chemotherapy within three days after implantation. Local anaesthesia was administered in the prospective operation area under sterile conditions and a 2–3 cm long skin incision was done 2–3 cm inferior of the clavicular base above the deltoid-pectoral region. After exhibition of the cephalic vein, the CVS technique was evaluated.

### Implantation of TIAP using guidewire with a hydrophilic coating (TVS, Figs. 2a-d)

**Fig. 2:**
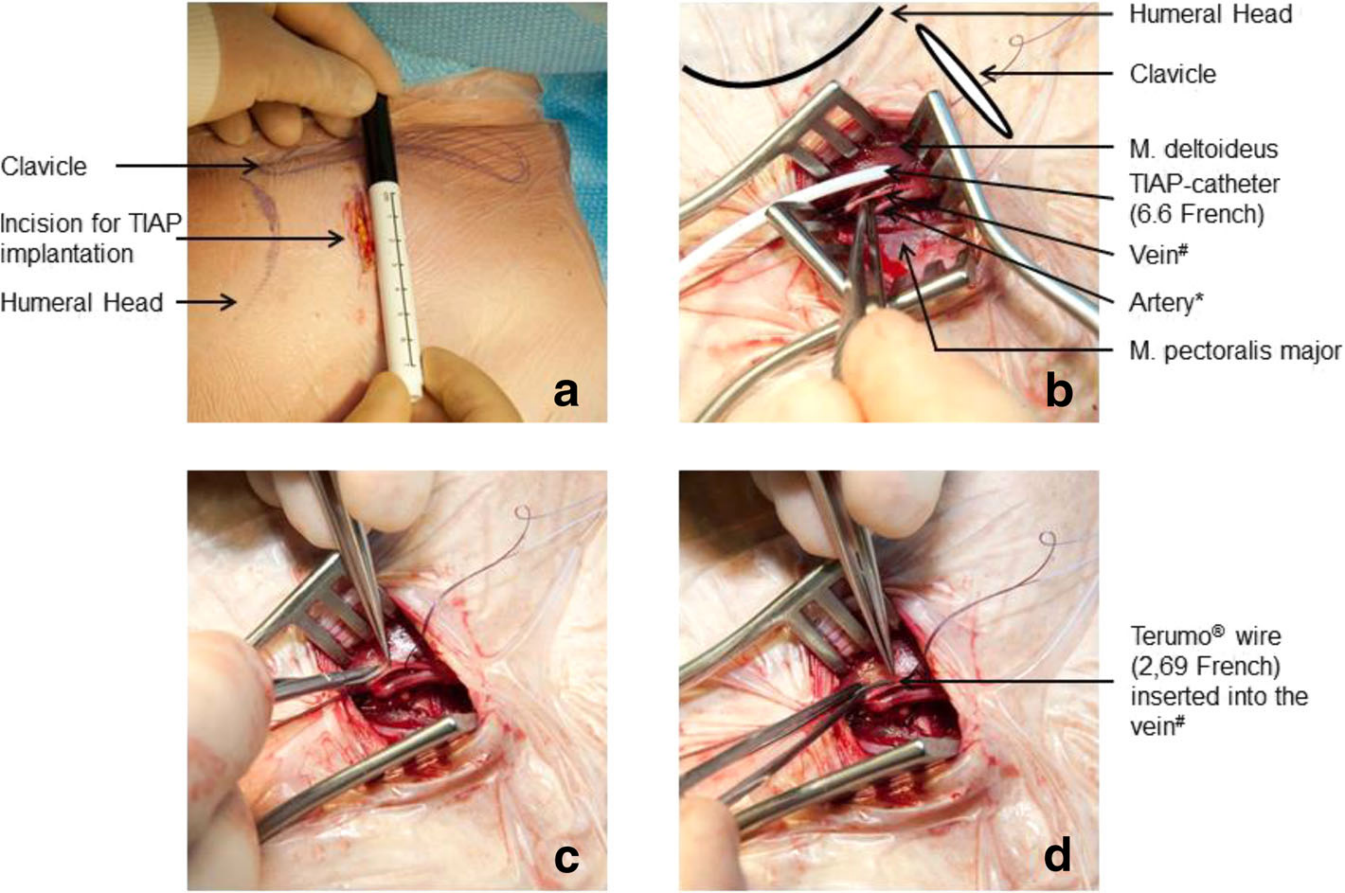
**a**-**d** Incision for Totally Implantable Access Port (TIAP) implantation according to: anatomical landmarks (**a**), with intraoperative situs (**b**), Vein cut-down/Venae Sectio using microsurgical instruments (**c**), and insertion of the guidewire with a hydrophilic coating (Terumo ® wire) (**d**). # Ramus pectoralis merging into V. thoracoacromialis. * Ramus pectoralis of A. thoracoacromialis

In cases where the cephalic vein was either non-existent or too small for CVS or MVS (Fig. 2b), the guidewire with a hydrophilic coating wire was used. The TVS started with ligation of the respective vessel (typically a small cephalic vein or Ramus pectoralis that merges into the V. thoracoacromialis) distally and encircled cranially with a 3–0 absorbable suture. With the use of magnification glasses and microsurgical instruments, a vein cut-down was performed (Fig. 2c) and the hydrophilic coated wire was inserted (Fig. 2d). Finally, the guide-wire was placed under fluoroscopy to the junction of the superior Vena Cava and right atrium. After positioning, either the TIAP catheter or a vein dilator and sheath were passed over the guide-wire. In cases where the latter was performed, the guide-wire and dilator were removed and the catheter was introduced through the peel-away sheath. After removal of the peel-away sheath, correct positioning of the implant was once again checked via fluoroscopy. Subsequently, the steps for port chamber connection and port chamber placement were done as per in-house standards (as described in previous publications) [22]. After CVP a routine x-ray was performed postoperatively to evaluate for pneumothorax.

### Objectives and outcomes

The primary aim was to establish whether or not the use of a guidewire with a hydrophilic coating reduced the number of punctures needed without correspondingly increasing complication rates. Also, the aim was to establish that neither the patients’ characteristics nor the surgeons’ experience play any significant role in the successful use of this guide-wire. Secondary aims of this study were to establish the independence of the results in cases where the operation was performed by an attending or a resident physician.

### Statistical analysis

The complication rates and the baseline characteristics were analysed with respect to the technique which was actually applied in each case. The success rate in the various treatment groups was determined and subsequently compared using the Kruskal-Wallis Test for continuous variables (Standard Deviation is provided in Tables 1 and 2) and Chi-square for dichotomous variables.

The rates of complications per year were calculated using a two-tailed chi-squared test. All analyses were performed using SPSS version 21.

## Results

In 2015, 1152 patients underwent operations that were classified as ‘Insertions of Intravenous Devices’. Overall, TIAP implantations were performed by 24 different surgeons, 9 of whom were board certified (data not shown). Out of the 1152 patients, 1099 patients actually received TIAP implantations, of which, the TIAP implantations were successfully performed via classical vein cut-down (CVS) in 822 cases (74.8%). Some 277 patients needed a second line rescue strategy, either via a primary Central Vein Puncture (CVP) or a modified vein cut-down using the hydrophilic coated guide-wire (TVS, Fig. 1 and Table 1). There were no statistically significant differences regarding demographics or indication for TIAP implantation (Table 1, *p* > 0.1, *p* - values not shown) between CVP and TVS.

**Fig. 3:**
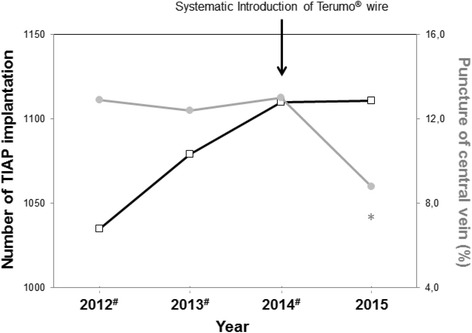
Number of Totally Implantable Access Port (TIAP) implantations (black boxes) and percentage of performed Central Vein Punctures (CVP) (grey circles) performed between 2012 and 2015. * The two-tailed *p* value is less than 0.0001 (as seen in a comparison of 2015 with 2014). # Up to December 2013, modified vein cut-down was performed by using a MHMedical Tec GmbH® guide-wire. The Terumo® wire was used sporadically in individual patients throughout 2014; however, it was introduced systematically in 2015

The operation time and the qualification of the operating surgeon between CVP and TVS showed no significant differences (Table 2). It was found that the use of the hydrophilic coated guide-wire as a second line rescue strategy reduced the need for CVP significantly from 12.7% to 8.8%(*p* < 0.0001, Fig. 3). Regarding early morbidity the incidence of pneumothorax was significantly higher in patients receiving CVP as a second line rescue strategy when compared to patients with TVS as a second line rescue strategy (*p* = 0.02, Table 2). There were no differences between CVP and TVS (Table 2) as it relates to late morbidity after TIAP implantation; this includes infection, non-function, and/or removal of TIAP.

## Discussion

### Safety of the TVS strategy

The data presented on the 277 patients who needed a second line rescue therapy during TIAP implantation allows the conclusion, that the use of the hydrophilic coated guide-wire as a second line rescue strategy significantly reduces not only the puncture rate during TIAP implantation, but also the incidence of pneumothorax. It is true that in experienced hands TIAP implantations can be performed safely whether or not CVP or CVS with its modifications are being used. Even though CVP includes a risk, per se, of developing of a pneumothorax, the published data reports this complication in about 1–3.2% of all cases [10, 26–28]. The reason to use primarily CVS and its modifications at our department is based on the fact that we generally perform more than 1000 TIAP implantations per year. For example, this means that even with a low, 3% incidence rate of pneumothorax after CVP, 30 of our patients would experience such complications on average. Furthermore, TIAP implantations are considered a reliable teaching procedure and will mostly be performed by residents during the early course of their training [27, 29].

As stated before, TIAP implantation using CVS (and its modifications) is not successful in all patients. It has been shown, under conditions of a randomized controlled trial, that TIAP implantation can be performed successfully in 92% of patients by using CVS [22]. However, the circumstances for TIAP implantation in our study could best be described as “daily routine”. Overall the frequency of complications is comparable to the existing literature and much lower in the case of thrombosis, suboptimal positioning or frequency of removal [29–32]. There has been a –not statistically significant- increased rate of infections in the study group which could not be due to the slightly increased operation time. According to an analysis conducted in our centre [19] some other reasons can account for this: ongoing chemotherapy, the female gender and breast cancer were recognized as independent and significant parameters. In the study group, there are more female patients with gynaecological malignancies, which could explain this finding.

Retrospectively seen, the need for CVP averaged about 12% throughout the years in our centre. This, together with the fact that an increasing number of patients with a need for recurrent TIAP implantation and patients with inadequately sized or even non-existent brachiocephalic vein, increases the need for appropriate and applicable modification techniques for CVS.

### Reproducibility and learning curve of the TVS strategy

At first glance, the process of TIAP implantation using TVS (as described in Fig. 1) seems technically demanding. However, the presented data has shown that the operation time for TIAP implantation between CVP and TVS do not differ significantly. In addition, 28.8% of the residents were able to apply this technique safely by themselves, while only 10.6% of cases required the support of an attending physician. This makes our findings, that TVS reduces significantly not only the puncture rate but also the incidence of pneumothorax even more clinically valuable. With this in mind, it would be interesting to examine the success and complication rates between CVP and SVS, for instance, in a multicentre expert-based setting.

## Conclusion

TIAP implantation using a guidewire with a hydrophilic coating significantly reduces the need for central vein puncture and the incidence of pneumothorax. TVS can be safely applied by surgeons at any stage of their training. Subsequently, it may even be beneficial to consider TVS as a first line technique for TIAP implantation.

## Abbreviations

CVP: Direct puncture of a central vein
CVS: Cut-down of the brachiocephalic vein
MVS: modified Seldinger technique (MVS)
TIAP: Totally Implantable Access Port
TVS: Terumo® Guide-wire as a second line rescue strategy
